# Spautin-1 inhibits mitochondrial complex I and leads to suppression of the unfolded protein response and cell survival during glucose starvation

**DOI:** 10.1038/s41598-022-15673-x

**Published:** 2022-07-07

**Authors:** Kazuhiro Kunimasa, Chika Ikeda-Ishikawa, Yuri Tani, Satomi Tsukahara, Junko Sakurai, Yuka Okamoto, Masaru Koido, Shingo Dan, Akihiro Tomida

**Affiliations:** 1 Division of Genome Research, Cancer Chemotherapy Center, Japanese Foundation for Cancer Research, Tokyo, 135-8550 Japan; 2 Division of Molecular Pharmacology, Cancer Chemotherapy Center, Japanese Foundation for Cancer Research, Tokyo, 135-8550 Japan; 3 Present Address: Division of Molecular Pathology, Institute of Medical Science, University of Tokyo, Tokyo, 108-8639 Japan

**Keywords:** Drug development, Cancer metabolism

## Abstract

The unfolded protein response (UPR) is an adaptive stress response pathway that is essential for cancer cell survival under endoplasmic reticulum stress such as during glucose starvation. In this study, we identified spautin-1, an autophagy inhibitor that suppresses ubiquitin-specific peptidase 10 (USP10) and USP13, as a novel UPR inhibitor under glucose starvation conditions. Spautin-1 prevented the induction of UPR-associated proteins, including glucose-regulated protein 78, activating transcription factor 4, and a splicing variant of x-box-binding protein-1, and showed preferential cytotoxicity in glucose-starved cancer cells. However, USP10 and USP13 silencing and treatment with other autophagy inhibitors failed to result in UPR inhibition and preferential cytotoxicity during glucose starvation. Using transcriptome and chemosensitivity-based COMPARE analyses, we identified a similarity between spautin-1 and mitochondrial complex I inhibitors and found that spautin-1 suppressed the activity of complex I extracted from isolated mitochondria. Our results indicated that spautin-1 may represent an attractive mitochondria-targeted seed compound that inhibits the UPR and cancer cell survival during glucose starvation.

## Introduction

Intracellular and extracellular stresses, such as oncogenic alternations and glucose starvation (GS), can lead to the accumulation of unfolded and misfolded proteins in the endoplasmic reticulum (ER)^1, 2^. This ER stress induces the unfolded protein response (UPR), which is initiated by three sensor proteins on the ER membrane: PKR-like ER kinase (PERK), inositol-requiring 1 (IRE1), and activating transcription factor 6 (ATF6)^2^. Upon the accumulation of abnormally folded proteins, the UPR sensor proteins activate various signaling cascades, including eukaryotic translation initiation factor 2A (eIF2α), to reduce global translation and induce stress-responsive transcriptional factors. ATF4 and the spliced form of x-box-binding protein-1 (XBP1s) are representative stress-responsive transcriptional factors and upregulate UPR-associated genes, such as the gene encoding the ER-resident molecular chaperone glucose-regulated protein 78 (GRP78). UPR proteins then alleviate the protein load and restore ER homeostasis, resulting in cell survival. In conditions of prolonged and unrecoverable stress, the UPR program leads to cell death via apoptosis^3^.

Cancer cells in solid tumors are often exposed to nutrient starvation and hypoxia that result from tumor vessels with structural and functional abnormalities and the huge energy demands for tumor growth^4^. Quantitative metabolome profiling has shown that glucose levels in tumor tissues are lower than those in adjacent normal tissues^5^. As GS can induce the UPR for cell survival, UPR-inhibiting molecules may represent promising candidates for chemotherapeutic agents. Our group and other groups have identified various UPR inhibitors, including versipelostatin, arctigenin, and biguanides (such as metformin, buformin, and phenformin)^6–9^. Some of these inhibitors have shown anti-cancer activity in vitro and in vivo and are being assessed in clinical trials^10, 11^.

In cancer cells under GS, impaired mitochondrial oxidative phosphorylation (OXPHOS) by mitochondrial complex inhibitors or depletion of mitochondrial DNA (mtDNA) results in failure of the UPR and massive cell death^9^. Furthermore, the growth of mtDNA-depleted cancer cells in a mouse xenograft model is markedly reduced, compared with that of mtDNA-intact cancer cells^12^. These findings indicate that functional mitochondria may be required for UPR activation and cancer cell survival under ER stress. ER stress augments mitochondria-ER contact and mitochondrial OXPHOS via a PERK-eIF2α-ATF4 axis for efficient cellular adaptation, suggesting critical roles of the inter-organelle crosstalk in the determination of the cell fate in response to ER stress^13–15^.

Specific and potent autophagy inhibitor-1 (spautin-1) is an autophagy inhibitor that suppresses the deubiquitination activity of ubiquitin-specific peptidase 10 (USP10) and USP13^16^. Inhibition of deubiquitinases by spautin-1 leads to ubiquitination and degradation of vacuolar sorting protein 34 (VPS34) and beclin-1, both of which are critical regulators of phagophore formation in early autophagy^17^. Spautin-1 has also shown antitumor activities alone or in combination with anti-cancer agents, such as doxorubicin and imatinib^18–22^. In screening for UPR inhibitors with potential anti-cancer activity, we found that spautin-1 inhibits the UPR and shows preferential cytotoxicity in glucose-starved cancer cells. We evaluated the mode of action of spautin-1 through transcriptomic and chemosensitivity-based COMPARE analyses and further showed that spautin-1 suppresses mitochondrial complex I activity. Our results suggest that spautin-1 may inhibit the UPR and cancer cell survival during glucose starvation through suppression of mitochondrial complex I activity.

## Results

### Spautin-1 inhibits the UPR and cancer cell survival under GS

**Figure 1:**
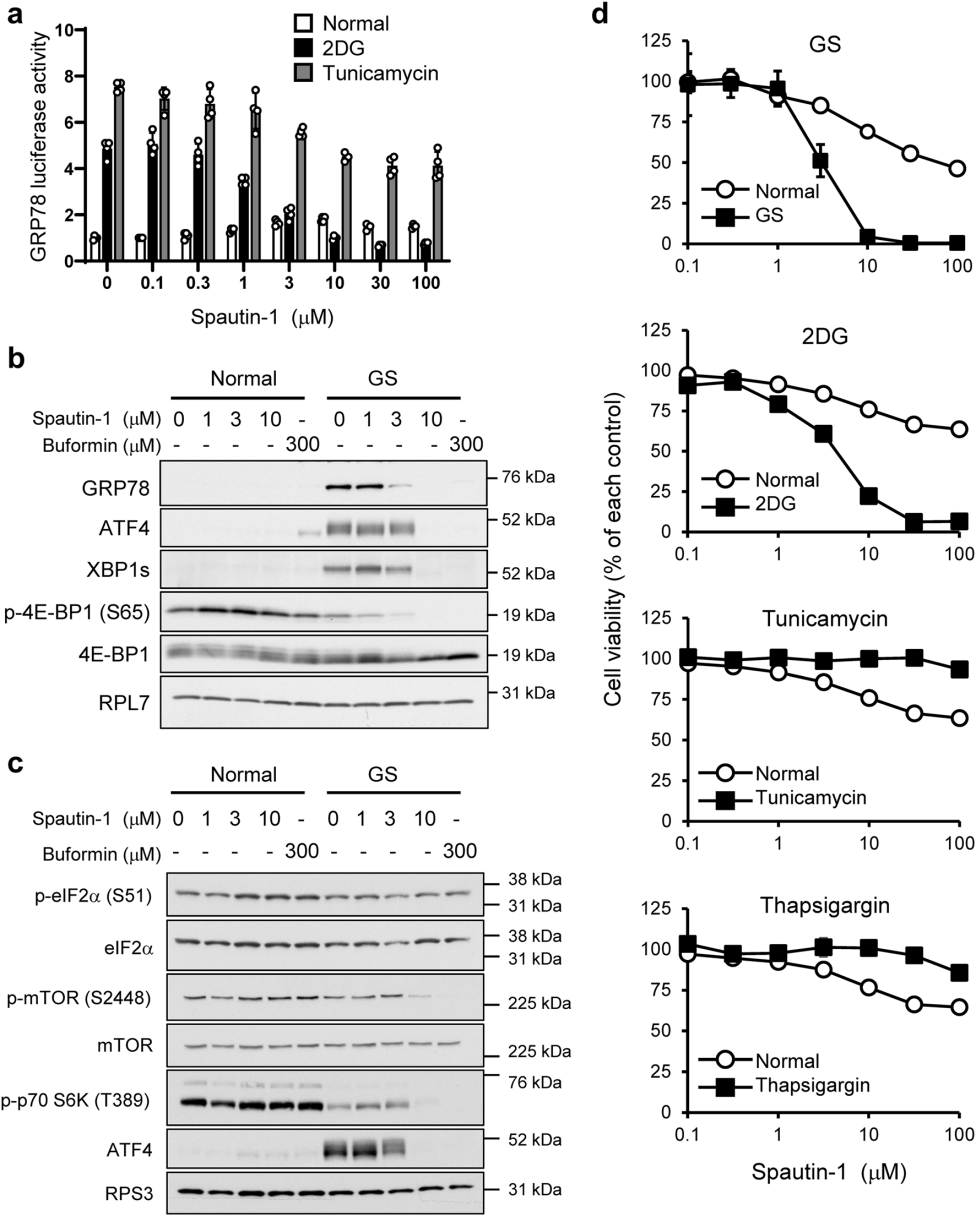
Spautin-1 inhibits the UPR and cell survival in glucose-starved and 2DG-stressed HT1080 cells. (**a**) HT1080 cells were transfected with the pGRP78pro160-Luc plasmid and then treated with spautin-1 in the presence of ER stressors (2DG: 10 mM, tunicamycin: 10 μg/mL) for 18 h. Luciferase activity was determined by a dual-luciferase reporter assay system. Data are shown as mean ± SD (*n* = 4). (**b**,**c**) HT1080 cells were treated with spautin-1 or buformin under control or GS conditions for 18 h, followed by western blot analysis for the indicated proteins. RPL7 and RPS3 were used as loading controls. The blot membranes were cut prior to hybridization with antibodies, according to Full range rainbow molecular weight markers. Original blots were presented in Supplementary Fig. S5. (**d**) HT1080 cells were treated with spautin-1 in the presence of ER stressors (2DG: 10 mM, tunicamycin: 10 μg/mL, thapsigargin: 300 nM) for 48 h. Cell viability was determined by the CellTiter-Glo luminescent cell viability assay. Data are shown as mean ± SD (*n* = 3).

As a system to evaluate activation of the UPR^7–9, 23–25^, we transfected human fibrosarcoma HT1080 cells with the GRP78-luc plasmid that contains the promoter region of *grp78* and exposed the cells to ER stressors, such as 2-deoxy-*D*-glucose (2DG, a glycolysis inhibitor and GS mimic) and tunicamycin (a *N*-glycosylation inhibitor). We found that spautin-1, an autophagy inhibitor, completely inhibited 2DG-induced promoter activity of GRP78 in HT1080 cells and only partially affected tunicamycin-induced luciferase activity (Fig. 1a).

To further evaluate the effects of spautin-1 on the UPR, we next investigated the expression of UPR-associated proteins under GS. Spautin-1 suppressed the induction of GRP78, ATF4, and XBP1s in glucose-starved HT1080 cells (Fig. 1b). Similar results were observed with buformin, a biguanide that prevents the UPR through inhibition of mitochondrial complex I activity^23, 26^. Spautin-1 also induced dephosphorylation of eIF4E-binding protein 1 (4E-BP1) in a concentration-dependent manner. The 4E-BP1 protein is a negative regulator of translation initiation and its hyperactivation by dephosphorylation is a useful marker associated with UPR inhibition in vitro and in vivo^23, 27^. Because ATF4 is a crucial factor for the UPR induction, we further investigated the effect of spautin-1 on the phosphorylation of eIF2α and mTOR, both of which regulate ATF4 induction under stress conditions^28, 29^. Spautin-1 had no or little effect on phosphorylation of eIF2α, while it as well as buformin suppressed the phosphorylation of mTOR and its downstream target, p70 S6K (Fig. 1c). These results indicated that spautin-1 may function as a novel UPR inhibitor under GS and that mTOR inhibition under GS by spautin-1 may be a possible mechanism of its UPR suppression.

We next compared the effects of spautin-1 on cell viability under control culture (glucose-rich culture) and GS conditions. Spautin-1 potently suppressed the cell viability of HT1080 cells (Fig. 1d) and various cancer cell lines under GS (Supplementary Fig. S1), while it had only limited effects under control culture conditions. We also evaluated the cytotoxicity of spautin-1 in the presence of chemical ER stressors, including 2DG, tunicamycin, and thapsigargin (a SERCA ATPase inhibitor) (Fig. 1d). Spautin-1 showed cytotoxicity in 2DG-stressed HT1080 cells but not in tunicamycin- and thapsigargin-stressed cells. The selectivity for GS and 2DG is similar to that observed with other UPR inhibitors, such as biguanides, mitochondrial complex inhibitors, and versipelostatin^7–9^.

### Spautin-1 suppresses the UPR transcriptional program under 2DG

**Figure 2:**
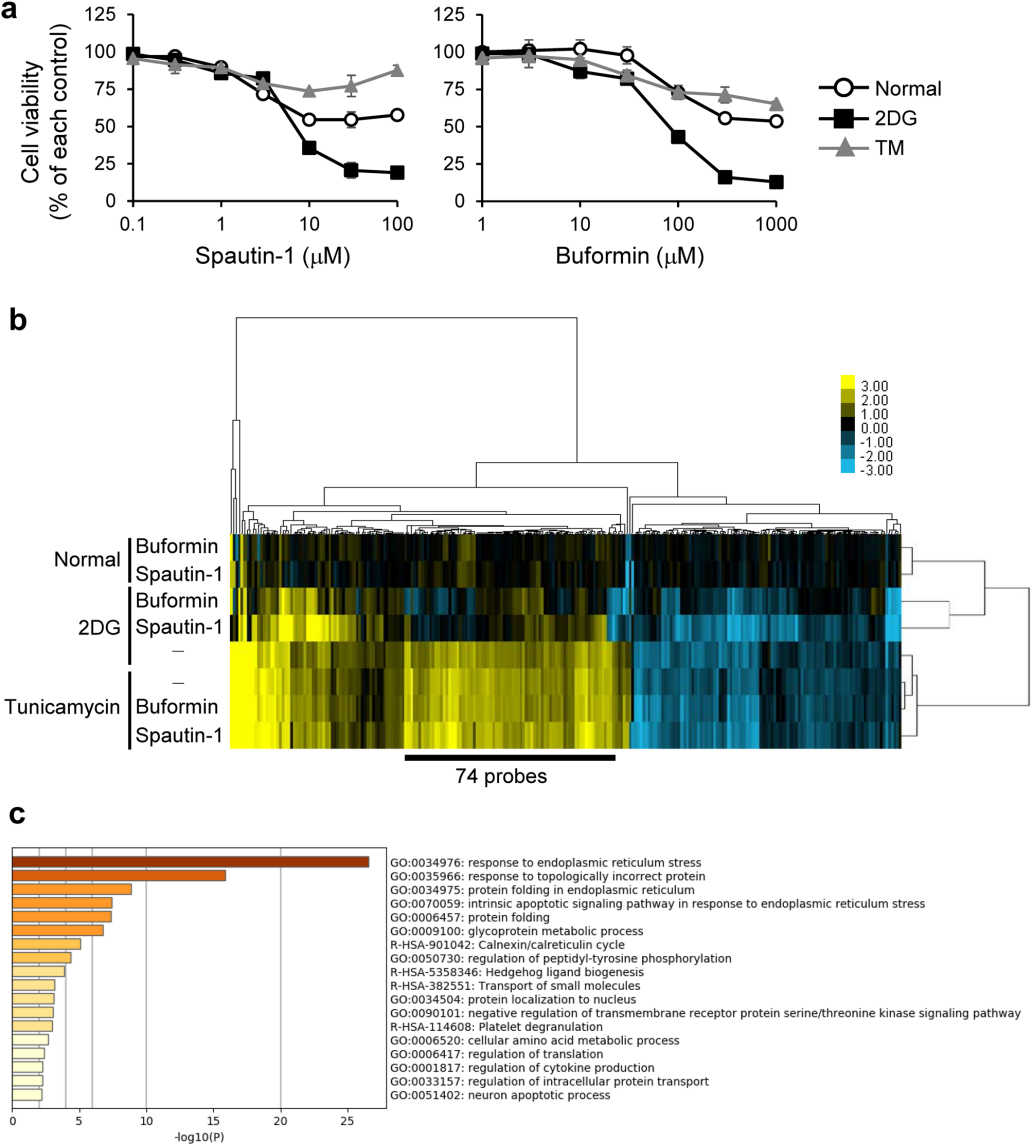
Spautin-1 suppresses the UPR transcriptional program in the presence of 2DG. (**a**) HT-29 cells were treated with spautin-1 or buformin in the presence of ER stressors (2DG: 10 mM, tunicamycin: 1 μg/mL) for 48 h. Cell viability was determined by the CellTiter-Glo luminescent cell viability assay. Data are shown as mean ± SD (*n* = 3). (**b**) HT-29 cells were treated with spautin-1 (10 µM) or buformin (300 µM) in the presence of ER stressors for 18 h. Changes in the UPR-associated expression signature (246 probe sets) were determined by DNA microarray and clustering analyses. (**c**) Gene Ontology analysis of the cluster (74 probe sets) that was upregulated by 2DG and downregulated by spautin-1 was analyzed by Metascape. The enriched terms with a *p* value < 0.01, a minimum count of 3, and an enrichment factor > 1.5 were listed.

Spautin-1 and buformin both showed cytotoxicity in HT-29 cells treated with 2DG (Fig. 2a), consistent with the above results. Using microarray-based transcriptome analyses, we previously identified a UPR-associated expression signature, consisting of 246 up or downregulated probes in GS- and 2DG-stressed HT-29 cells and other cancer cell lines^7^. We next evaluated the effects of spautin-1 and buformin on the UPR expression signature. Both compounds similarly counteracted 2DG-induced but not TM-induced changes in the UPR expression signature (Fig. 2b and Supplementary Tables S1, S2). These results were consistent with the preferential cytotoxicity of spautin-1 in HT1080 and HT-29 cells treated with 2DG but not TM (Figs. 1d, 2a). Gene Ontology (GO) analyses by the functional annotation tools Metascape and DAVID revealed that GO terms on the UPR and response to ER stress (Fig. 2c and Supplementary Table S3, S4) were enriched in the downregulated cluster (74 probe set, Supplementary Table S5), indicating that spautin-1 suppresses the UPR transcriptional program in the presence of 2DG.

### USP10 and USP13 silencing has little effect on the UPR under GS

**Figure 3:**
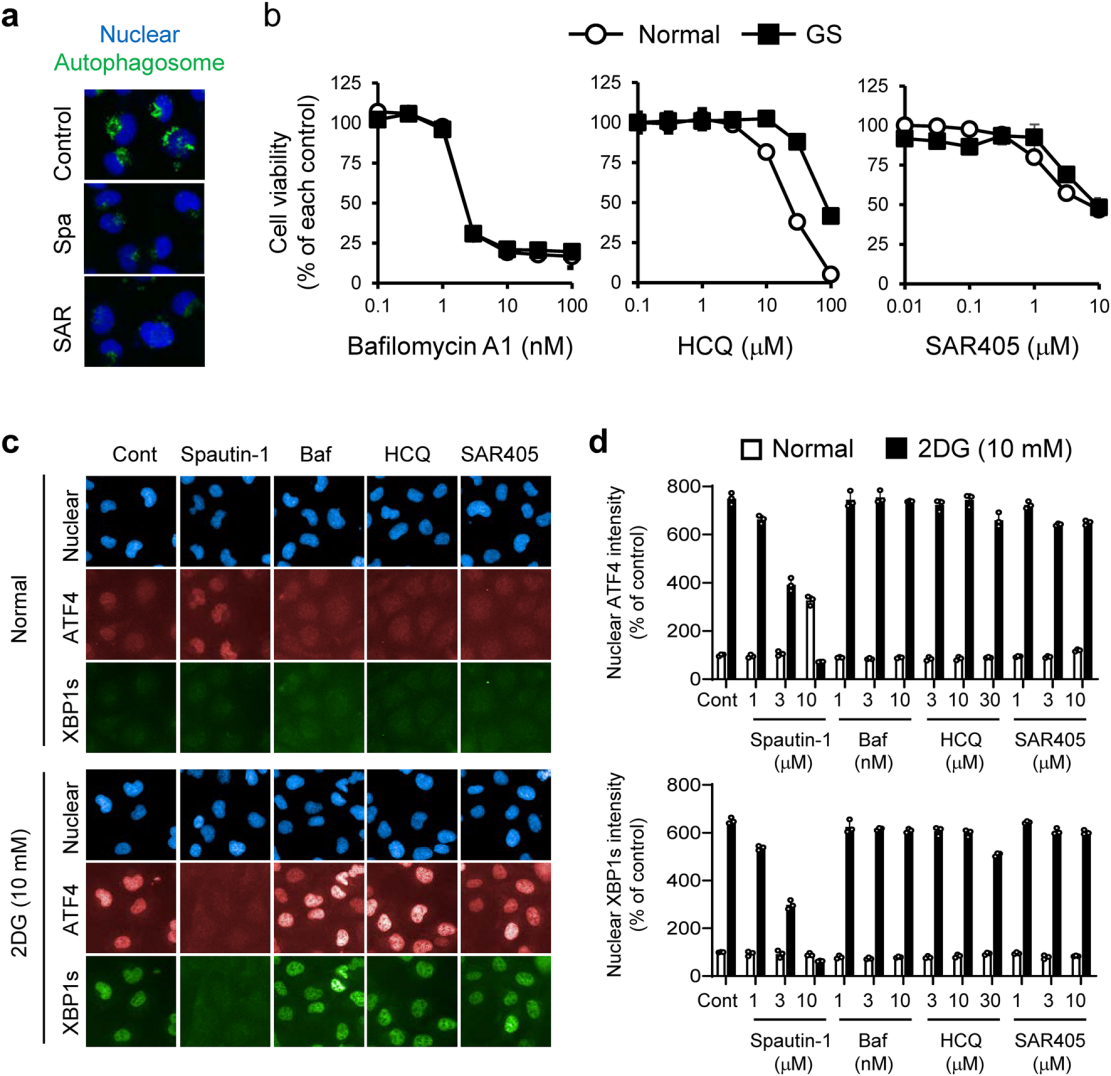
Autophagy inhibitors fail to show preferential cytotoxicity and suppress induction of ATF4 and XBP1s in glucose-starved or 2DG-stressed HT1080 cells. (**a**) HT1080 cells were treated with spautin-1 (10 μM) or SAR405 (1 μM) in HBSS containing HCQ (30 μM) for 4 h. Autophagosomes were visualized with the CYTO-ID Autophagy detection kit 2.0. (**b**) Effects of bafilomycin A1 (Baf), HCQ, and SAR405 on cell viability in GS-stressed HT1080 cells were determined by the CellTiter-Glo luminescent cell viability assay. Data are shown as mean ± SD (*n* = 3). (**c**) Effects of spautin-1 (10 μM) and other autophagy inhibitors (Baf; 10 nM, HCQ; 30 μM, SAR405; 10 μM) on nuclear ATF4 and XBP1s induction under 2DG-stressed conditions were visualized using the Operetta CLS. Blue, red, and green fluorescent signals indicate nuclei, ATF4, and XBP1s, respectively. (**d**) Mean intensities of nuclear ATF4 and XBP1s intensities were determined using Harmony high-content analysis software. Data are shown as mean ± SD (*n* = 3).

Previous studies showed that spautin-1 inhibits autophagy^16^. Consistent with these findings, we found that spautin-1 suppressed autophagosome formation under Hanks' balanced salt solution (HBSS) in HT1080 cells (Fig. 3a and Supplementary Figure S2a,b). We next investigated the role of autophagy in spautin-1-mediated inhibition of UPR using other autophagy inhibitors, such as SAR405, bafilomycin A1, and hydroxychloroquine sulfate (HCQ). SAR405 is a kinase inhibitor of VPS34, a PI3K class III isoform, that is complexed with beclin-1 at the early stage of autophagy and promote autophagy^30^. Bafilomycin A1 and HCQ prevent fusion of autophagosome and lysosome at the late stage of autophagy. In contrast to spautin-1, SAR405, bafilomycin A1, and HCQ failed to exhibit preferential cytotoxicity under GS (Fig. 3b). Furthermore, the three inhibitors had no effect on ATF4 and XBP1s induction in 2DG-stressed HT1080 cells (Fig. 3c,d). These results implied that the preferential cytotoxicity and UPR inhibition of spautin-1 under GS- or 2DG-stressed conditions are independent of autophagy inhibition.

**Figure 4:**
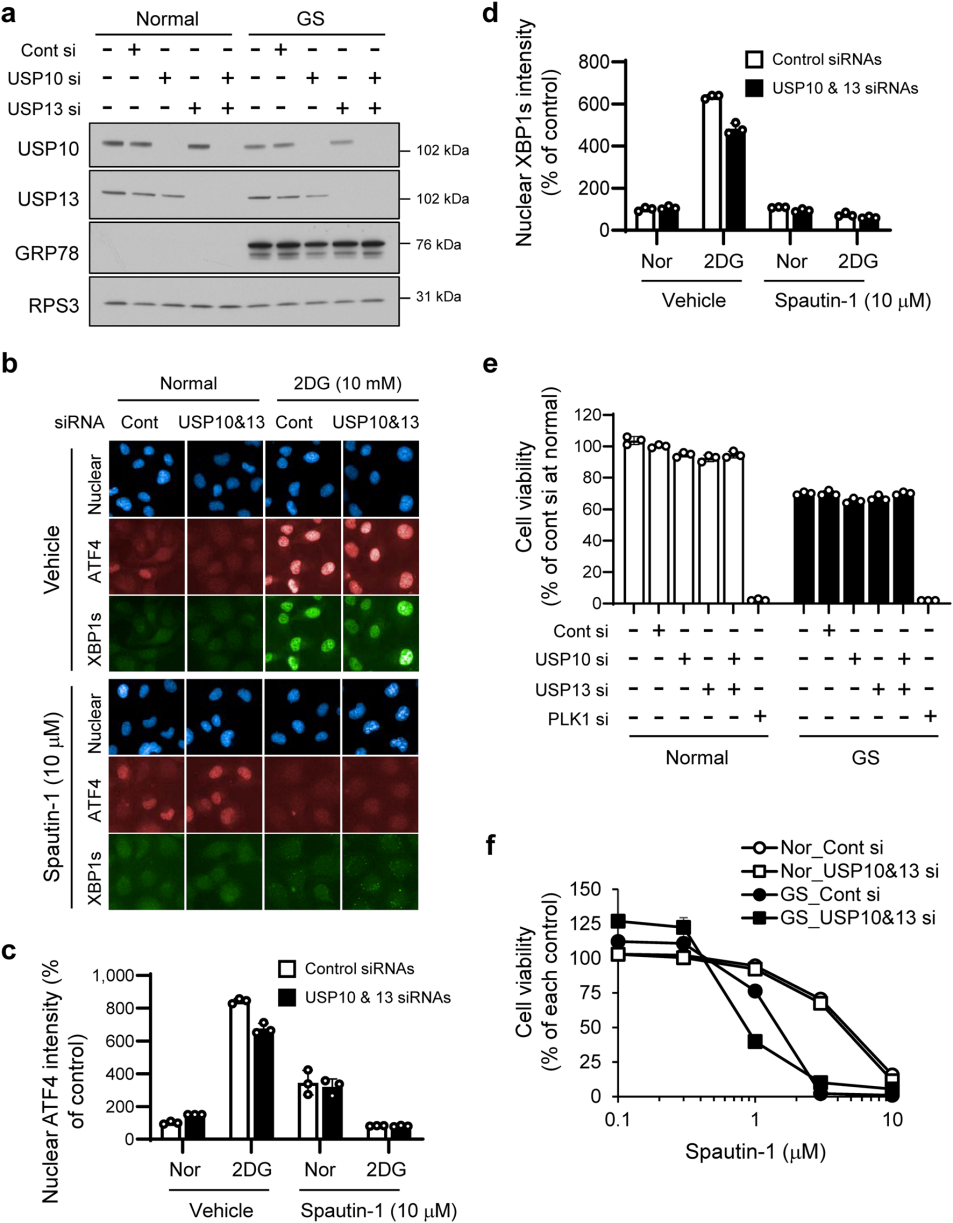
USP10 and USP13 silencing has little effect on the UPR and cell viability under GS- or 2DG-stressed conditions. (**a**) Effects of USP10 and USP13 silencing on GRP78 in HT1080 cells were determined by western blotting. RPS3 was used as a loading control. The blot membranes were cut prior to hybridization with antibodies, according to Full range rainbow molecular weight markers. Original blots were presented in Supplementary Fig. S5. (**b**) Effects of USP10 and USP13 silencing on ATF4 and XBP1s induction in vehicle- or spautin-1-treated HT1080 cells under 2DG-stressed conditions were visualized using the Operetta CLS. (**c**,**d**) Mean intensities of nuclear (**c**) ATF4 and (**d**) XBP1s in (**b**) were determined using Harmony high-content analysis software. Data are shown as mean ± SD (*n* = 3). (**e**) Effects of USP10 and USP13 silencing on cell viability under GS were determined by the CellTiter-Glo luminescent cell viability assay. Data are shown as mean ± SD (*n* = 3). (**f**) Effects of USP10 and USP13 silencing on preferential cytotoxicity of spautin-1 under GS were determined by the CellTiter-Glo luminescent cell viability assay. Data are shown as mean ± SD (*n* = 3).

The USP10 and USP13 deubiquitinases have been identified as molecular targets of spautin-1^16^. Silencing of USP10 and USP13 by siRNAs resulted in a reduction in 2DG-induced autophagy (Supplementary Fig. S2c,d). We next investigated the possible involvement of USP10 and USP13 suppression in the preferential cytotoxicity and UPR inhibition of spautin-1. Knockdown of USP10, USP13, or both in HT1080 cells had little effect on the induction of UPR markers, GRP78, ATF4, and XBP1s under GS- or 2DG-stressed conditions (Fig. 4a–d). Cell survival of USP10- and/or USP13-knocked-down HT1080 cells under GS was comparable with that of the control siRNAs-transfected cells (Fig. 4e). We further investigated the effects of USP10 and USP13 silencing on autophagy suppression (Supplementary Fig. S2c,d), preferential cytotoxicity (Fig. 4f), and UPR inhibition (Fig. 4b–d) of spautin-1. Simultaneous silencing of both USP10 and USP13 had no or little effect on the inhibitory effects of spautin-1. These findings indicated that spautin-1-mediated preferential cytotoxicity and inhibition of UPR is independent of USP10 and USP13 in our experimental conditions.

### Spautin-1 is a novel mitochondrial complex I inhibitor

**Figure 5:**
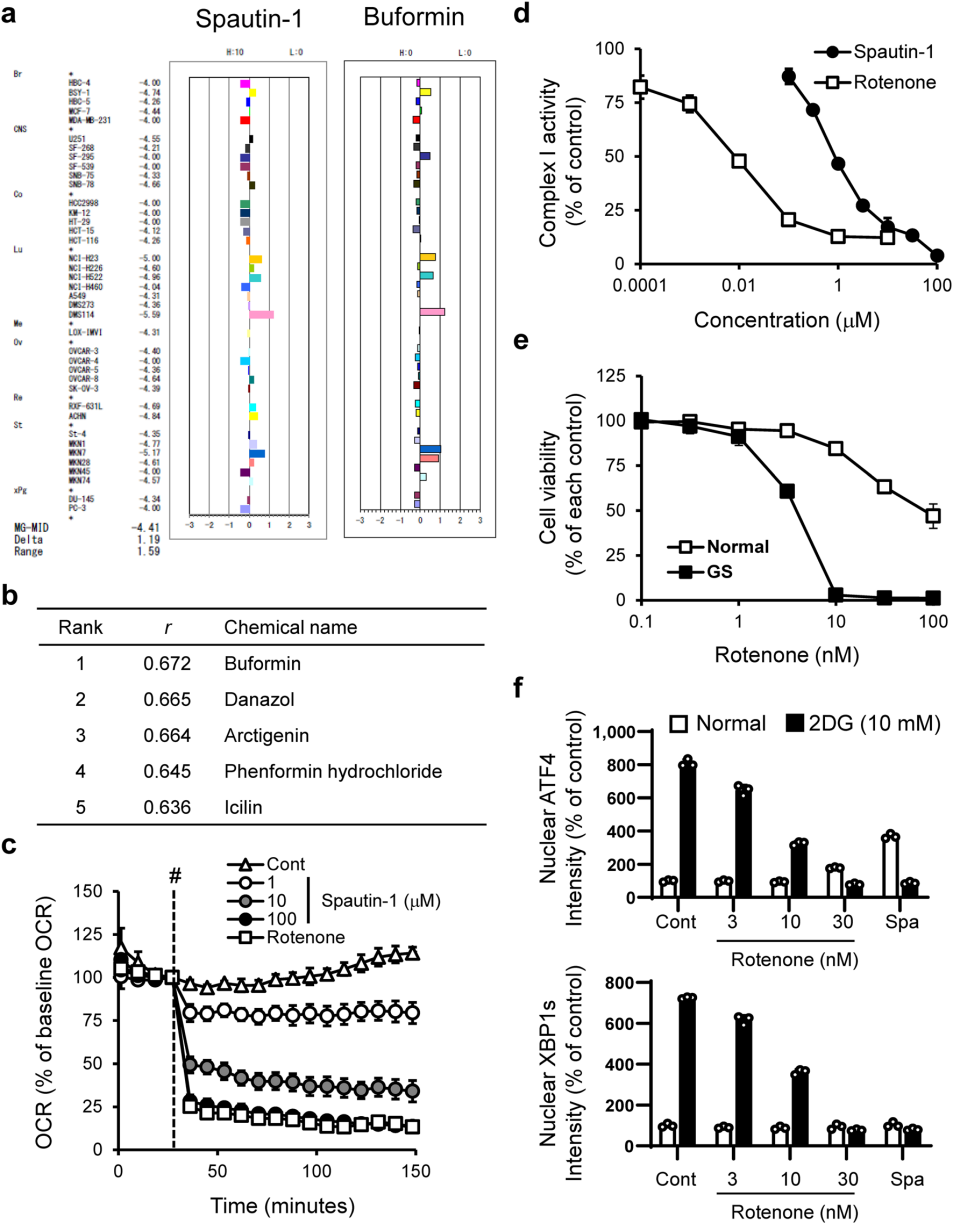
Spautin-1 inhibits mitochondrial complex I activity**.** (**a**) The mean graph was produced by computer processing of the 50% growth inhibition (GI50) values. The mean graph of spautin-1 (left) is very similar to that of buformin (right). Bottom left: MG-MID, the mean of log GI50 values for 39 cell lines; Delta, the logarithm of difference between the MG-MID and the log GI50 of the most sensitive cell line; range, the logarithm of difference between the log GI50 of the most resistant cell line and the log GI50 of the most sensitive cell line. (**b**) COMPARE analysis between the chemical fingerprints of spautin-1 and anticancer compounds in the JFCR39 cancer cell line panel was performed. (**c**) Oxygen consumption rate (OCR) in HT1080 cells was measured by the XF extracellular flux analyzer. OCR indicates the rate of mitochondrial respiration. Rotenone (100 nM), a mitochondrial complex I inhibitor, was used as a positive control. Data are shown as mean ± SD (*n* = 4). (**d**) Effect of spautin-1 on mitochondrial complex I was determined using the MitoTox Complex I OXPHOS Activity kit. Data are shown as mean ± SD (*n* = 3). (**e**) Effect of rotenone on cell viability under GS was determined by the CellTiter-Glo luminescent cell viability assay. Data are shown as mean ± SD (*n* = 3). (**f**) Effects of rotenone and spautin-1 (10 μM) on induction of nuclear ATF4 and XBP1s under 2DG-stressed conditions were determined using Harmony high-content analysis software. Data are shown as mean ± SD (*n* = 3).

To investigate the mode of action of spautin-1, we used the JFCR39 human cancer cell panel consisting of 39 cell lines from various tissues and performed COMPARE analysis using fingerprints of spautin-1 and JFCR39 drug database (Fig. 5a). Pearson correlation coefficient (*r*) between the GI50 mean graphs of spautin-1 and the reference compounds was calculated. The* r* value of more than 0.6 between two agents suggests they might have a similar action mechanism^31, 32^. The COMPARE analysis suggested that the possible mode of action of spautin-1 is similar to that of biguanides, such as buformin and phenformin hydrochloride (Fig. 5b). The compounds listed in Fig. 5b, except for icilin, have been reported to inhibit mitochondrial complex activity^26, 33–35^.

Mitochondrial function is indispensable for cancer cell survival under GS^9^. Biguanides inhibit the UPR and cancer cell killing under GS by suppressing mitochondrial complex I activity^23, 26^. To evaluate the effect of spautin-1 on mitochondrial function, we performed an extracellular flux analysis. Spautin-1 reduced the oxygen consumption rate (OCR), an indicator of mitochondrial respiration (Fig. 5c). We further investigated the effect of spautin-1 on mitochondrial complex I activity using complex I extracted from isolated mitochondria. In line with the above results, spautin-1 suppressed mitochondrial complex I activity in a concentration-dependent manner (Fig. 5d). Furthermore, we used rotenone, a well-defined mitochondrial complex I inhibitor, to support a mechanistic link between the UPR inhibition and mitochondrial complex I suppression. Rotenone showed preferential cytotoxicity and suppressed induction of ATF4 and XBP1s under GS- or 2DG-stressed conditions (Fig. 5e,f, Supplementary Fig. S3). These findings indicated that spautin-1 may inhibit the UPR and cancer cell survival under GS- and 2DG-stressed conditions through suppression of mitochondrial complex I activity.

## Discussion

Spautin-1 has been reported as an USP10- and USP13-targeting autophagy inhibitor^16^. Our results showed that spautin-1 inhibits the UPR under GS- and 2DG-stressed conditions. However, autophagy inhibition and USP10 and USP13 silencing had little or no effect on the UPR and cell survival in glucose-starved HT1080 cells. These results indicate that spautin-1 exhibits the UPR inhibition and preferential cytotoxicity in an autophagy- and USP10/USP13-independent manner. Laio et al. reported that spautin-1 induces cell cycle arrest and apoptosis of prostate cancer cell lines in a USP10/USP13-independent manner^36^. These findings led us to more closely investigate the potential mode of action of spautin-1. Cell line-based platforms, such as JFCR39 and National Cancer Institute 60, have helped predict and uncover novel targets of various types of anti-cancer agents^37–40^. We used the JFCR39 panel system and our results demonstrated that spautin-1 is a novel OXPHOS inhibitor.

Silencing of USP10 and USP13 caused a reduction in autophagosome formation under 2DG-stressed conditions (Supplementary Fig. S2c,d), which were accompanied with a slight decrease in protein expression levels of beclin-1 (Supplementary Fig. S4b), a target of USP10 and USP13 deubiquitination^16^. These findings indicates that USP10 and USP13 can be functional to regulate autophagy and beclin-1 degradation in HT1080 cells. On the other hand, we found that spautin-1 inhibited autophagosome formation in HT1080 cells but did not observe any effects on the protein levels of VPS34 and beclin-1 in our experimental conditions (Supplementary Fig. S4a,b). Recent studies showed that spautin-1 suppresses autophagy without affecting VPS34 and beclin-1 levels^21, 41^. These findings suggested that the involvement of VPS34 and beclin-1 in autophagy inhibition by spautin-1 depends on the environmental and cellular context. In contrast, other groups reported that mitochondrial complex I inhibitors, including biguanides, aumitin, and authipyrin, suppress nutrient starvation- and mTOR inhibitor-induced autophagy^42–44^. Furthermore, knockout cell lines of NDUFA1 and NDUFA10, mitochondrial complex I accessory subunits, showed severe autophagy defects^42^. The previous studies proposed that mitochondrial complex I inhibition causes autophagy suppression through decreases in mitochondria-associated membranes and mitochondrial phosphatidylethanolamine biosynthesis. Given that ER-mitochondria contact increases in the early phase of ER stress^15^, it might be important to clarify the potential influence of spautin-1 on the inter-organelle contact and crosstalk in future studies.

Clinical development of OXPHOS inhibitors has been challenging, due to a lack of predictive biomarkers for efficacious responses to inhibitors of this biological process. However, recent advances in transcriptomic and metabolic profiling highlight OXPHOS dependency even under nutrient-rich conditions in certain tumors, including acute myeloid leukemia, pancreatic cancer, and glioma^45–48^. Intense efforts in the development of OXPHOS inhibitors has led to the identification of promising candidates, such as IACS-010759, Gboxin, and mubritinib^46–48^. IACS-010759, with tolerable side effects, is being evaluated in advanced solid tumors and acute myeloid leukemia in clinical trials. Furthermore, advanced PET probes and contrast agents for MRI that can detect OXPHOS activity in tumors were developed and enable the in vivo evaluation of OXPHOS inhibitors^49, 50^. These attempts further the development of OXPHOS inhibitors. Additional evaluation and optimization on spautin-1 are needed for clinical application.

In conclusion, here we demonstrated that spautin-1 is a novel and potent OXPHOS inhibitor that can suppress cell viability of various types of cancer cell lines and the UPR under GS. Because chemical structure of spautin-1 is distinct from those of other OXPHOS inhibitors, including rotenone, biguanides, and IACS-010759, spautin-1 may represent a promising seed compound for the development of tumor microenvironment-selective chemotherapeutic agents.

## Methods

### Cell culture and reagents

HT1080 human fibrosarcoma cells were obtained from the American Type Culture Collection (Manassas, VA, USA). The JFCR39 human cancer cell line panel was developed and cultured as described previously^31^. HT1080 cells and the JFCR39 cell lines were maintained in RPMI1640 (FUJIFILM Wako Pure Chemical Industry, Osaka, Japan) supplemented with 10% or 5% fetal bovine serum (FBS) and 100 μg/mL kanamycin, unless otherwise stated. Spautin-1, bafilomycin A1, HCQ, SAR405, and rotenone were purchased from Selleck Chemicals (Houston, TX, USA) and dissolved in DMSO. We obtained 2DG from Sigma-Aldrich (St Louis, MO, USA) and 2DG was dissolved in sterilized distilled water. Tunicamycin (Nacalai Tesque, Kyoto, Japan) and thapsigargin (FUJIFILM Wako Pure Chemical Industry) were dissolved in DMSO.

### GRP78 luciferase reporter assay

HT1080 cells were transfected with a firefly luciferase gene-containing reporter plasmid (pGRP78pro160-Luc) that contains human *grp78* promoter region (nucleotides − 160 to + 7 to the start of transcription) and the Renilla luciferase gene-containing plasmid phRL-CMV (Promega, Madison, WI, USA) as an internal control, as described previously^8, 25^. The transfected cells were treated with spautin-1, 2DG (10 mM), and/or TM (10 μg/mL) for 18 h. The cells were harvested and assayed using the Dual-Glo Luciferase Assay System (Promega), and the relative activity of firefly luciferase to Renilla luciferase was determined.

### RNA interference

Silencing of human USP10 and USP13 expression was performed using Dharmacon ON-TARGETplus SMARTpool siRNAs (USP10: L-006032-00-0010; USP13: L-006064-00-0010, Horizon Discovery, Cambridge, United Kingdom). The non-targeting control pool (D-001810-10-50, Horizon Discovery) was used as control. The siRNAs (20 nM) were transfected using Lipofectamine RNAiMAX transfection reagent (Thermo Fisher Scientific) in accordance with the manufacturer’s reverse transfection protocol. After 24 h or 48 h, the cells were used for further experiments. Because PLK1 knockdown caused severe cell death, PLK1 siRNAs (L-003290-00-0005, Horizon Discovery) was used as an experimental control for transfection.

### Immunoblot analysis

Immunoblot analysis was performed as described previously^28^. Briefly, cells were lysed in SDS lysis buffer and protein concentrations were determined using the Bio-Rad protein assay (Bio-Rad, Hercules, CA, USA). Protein samples and full range rainbow molecular weight markers RPN800E (Cytiva, Marlborough, MA, USA) were separated by SDS-PAGE and transferred onto a nitrocellulose membrane. Before hybridization with antibodies, nitrocellulose membranes were cut with at least one molecular weight marker, according to the RPN800E molecular weight markers. The membranes were immunoblotted with the following primary antibodies: anti-ATF4 (Cat#: 11815), anti-XBP1s (Cat#: 12782), anti-phospho-4E-BP1 (Ser65) (Cat#: 9451), anti-4E-BP1 (Cat#: 9452), anti-USP10 (Cat#: 8501), anti-USP13 (Cat#: 12577), anti-VPS34 (Cat#: 4263), anti-beclin-1 (Cat#: 3495), anti-phospho-mTOR (Ser2448) (Cat#: 2971), anti-mTOR (Cat#: 2972), anti-phospho-p70 S6K (Thr389) (Cat#: 9234), anti-phospho-eIF2α (Ser51) (Cat#: 3398), anti-RPL7 (Cat#: 2403), anti-RPS3 (Cat#: 9538) (all from Cell Signaling Technology, Danvers, MA, USA), anti-eIF2α (Cat#: ab5369; Abcam, Cambridge, United Kingdom), and anti-KDEL, an antibody that recognizes GRP78 (Cat#: ADI-SPA-827; Enzo Life Sciences, Farmingdale, NY, USA). The specific bands were detected using Western Lightning plus ECL (Perkin Elmer, Waltham, MA, USA) and the X-OMAT 2000 film processor (Kodak, Rochester, NY, USA) or Amersham ImageQuant 800 (Cytiva). Original scan images for western blots are presented in Supplementary Fig. 5.

### Cell viability assay

Cells were seeded at 5 × 10^3^ (HT1080, HT-29, HBC-4, PC-3, RXF-631L, and SK-OV-3) and 1 × 10^4^ (MKN74) cells/well and cultured for 24 h. For siRNA experiment, HT1080 cells were transfected with control, USP10, USP13, or combination of USP10 and USP13 siRNAs and cultured for 24 h. The cells were treated with vehicle (DMSO), spautin-1, bafilomycin A1, HCQ, SAR405, or rotenone in normal (glucose-rich) or glucose-free RPMI1640 (Thermo Fisher Scientific, Waltham, WA, USA) with 10% FBS for 48 h. Cell viability was determined by the CellTiter-Glo luminescent cell viability assay (Promega) and quantification of the ATP content in accordance with the manufacturer’s protocol.

### Microarray analysis

HT-29 cells were incubated with spautin-1 (10 μM) or buformin (300 μM) under vehicle (DMSO), 2DG (10 mM), and tunicamycin (1 μg/mL) treatment for 18 h. Total RNA was purified using the RNeasy RNA purification kit (Qiagen, Hilden, Germany). The quality of total RNA was analyzed using the RNA 6000 Nano LabChip kit (Agilent Technologies, Santa Clara, CA, USA) on an Agilent 2100 Bioanalyzer. Microarray analysis was carried out using the Affymetrix GeneChip Human genome U133 Plus 2.0 Array (Affymetrix, Santa Clara, CA, USA). Normalization of microarray data was carried out by the MAS5. Probes with low (less than 50) signal intensity in all arrays were treated as a fixed value of 50. The log ratio for each gene was calculated by setting the expression level in the appropriate control sample as 0 (log_2_).

We defined the UPR-associated expression signature consisting of 246 probe sets with the fold change cutoff of > 2 fold up and down under GS or 2DG compared with control condition^7^. The lists of probe sets are provided in Supplementary Table 1 (148 upregulated probes) and 2 (96 downregulated probes).

Clustering analysis was performed by average linkage analysis using Cluster 3.0 software and visualized by Java TreeView. Gene Ontology analyses were performed using Metascape (http://metascape.org, release 3.5)^51^ and DAVID (https://david.ncifcrf.gov/, DAVID 6.8)^52^. In the Metascape analysis, the enrichment terms with a *p* value < 0.01, a minimum count of 3, and an enrichment factor > 1.5 (the enrichment factor is the ratio between the observed counts and the counts expected by chance) were collected and listed.

### Autophagy imaging

HT1080 cells were seeded at 8 × 10^3^ cells/well in a 96-well plate. After 24 h incubation, the cells were treated for 4 h with vehicle (DMSO), spautin-1 (10 μM), or SAR405 (1 μM) in HBSS containing HCQ (30 μM), which was used to stop autophagic flux. Nuclei and autophagosomes were stained with Hoechst33342 and CYTO-ID green detection reagent 2 from the CYTO-ID Autophagy detection kit 2.0 (Enzo Life Sciences) for 30 min. Autophagosomes were observed using the IN Cell Analyzer 6000 (Molecular Devices, San Jose, CA, USA).

### Fluorescent immunostaining

HT1080 cells were plated on CellCarrier-96 Ultra (Perkin Elmer) at 8 × 10^3^ cells/well and treated with spautin-1, HCQ, bafilomycin A1, SAR405, or rotenone in the presence or absence of 2DG (10 mM) for 4 h. For siRNA experiments, HT1080 cells were transfected with control siRNAs or combination of USP10 and USP13 siRNAs. After 48 h incubation, the cells were treated with vehicle or spautin-1 (10 μM) under control or 2DG-stressed conditions for 4 h. The cells were subjected to fluorescent immunostaining using anti-ATF4 rabbit monoclonal antibody (Cat#: 11815, Cell Signaling Technology), anti-XBP1s mouse monoclonal antibody (Cat#: 27901, Cell Signaling Technology), Alexa Flour Plus 647 goat anti-rabbit IgG secondary antibody for ATF4 (Cat#: A32733, Thermo Fisher Scientific), Alexa Flour Plus 488 goat anti-mouse IgG secondary antibody for XBP1s (Cat#: A32723, Thermo Fisher Scientific), and nuclear staining with Hoechst 33342, as described previously^25^. Fluorescent images (nine fields per well) were acquired using a 20 × water objective lens by Operetta CLS (Perkin Elmer). Quantification of ATF4 and XBP1s mean intensities in nuclei was performed using Harmony high-content analysis software (Version 4.9, Perkin Elmer).

### COMPARE analysis

COMPARE analysis using the JFCR39 panel was performed as described previously^31, 32^. Briefly, after 48 h of drug treatment, the inhibition of cell proliferation was assessed by use of a sulforhodamine B assay that measures changes in total cellular proteins. The 50% growth inhibition (GI_50_) value was calculated as described previously^31, 32^. Using these sets of GI_50_ values, fingerprints are presented in the graphic profiles of relative sensitivity within JFCR39. To analyze the correlation between the fingerprints of drug A and drug B, we used the COMPARE computer algorithm. The Pearson correlation coefficient (*r*) between the fingerprints of drug A and drug B was calculated (*n* = 39).

### OCR measurement

OCR was determined using an XF24 Extracellular Flux Analyzer (Seahorse Bioscience, North Billerica, MA, USA), as described previously^12^. We defined the OCR of normal (non-stressed) condition at the fourth measurement time point as the baseline OCR.

### Mitochondria complex I activity

Mitochondrial complex I activity was determined using the MitoTox Complex I OXPHOS activity microplate assay kit (Abcam), in accordance with the manufacturer’s instructions.

## Supplementary Information

Supplementary Information 1.

Supplementary Information 2.

## Data Availability

The accession number for the microarray data reported in this paper is National Center for Biotechnology Information Gene Expression Omnibus: GSE189850.
