# Supplementary material for: Spautin-1 inhibits mitochondrial complex I and leads to suppression of the unfolded protein response and cell survival during glucose starvation
Source: Sci Rep. 2022 Jul 7;12:11533. doi: 10.1038/s41598-022-15673-x (PMC9262966; doi:10.1038/s41598-022-15673-x)

Spautin-1 inhibits mitochondrial complex I and leads to suppression of the unfolded protein response and cell survival during glucose starvation

Kazuhiro Kunimasa[1], Chika Ikeda-Ishikawa[1], Yuri Tani[1], Satomi Tsukahara[1], Junko Sakurai[1], Yuka Okamoto[1], Masaru Koido[1,2], Shingo Dan[3], and Akihiro Tomida[1,*]

[1]Division of Genome Research, Cancer Chemotherapy Center, Japanese Foundation for Cancer Research, Tokyo 135-8550, Japan. [2]Present address: Division of Molecular Pathology, Institute of Medical Science, University of Tokyo, Tokyo 108-8639, Japan. [3]Division of Molecular Pharmacology, Cancer Chemotherapy Center, Japanese Foundation for Cancer Research, Tokyo 135-8550, Japan. *Correspondence and requests for materials should be addressed to A.T. (email: akihiro.tomida@jfcr.or.jp)

Supplementary Figure S1

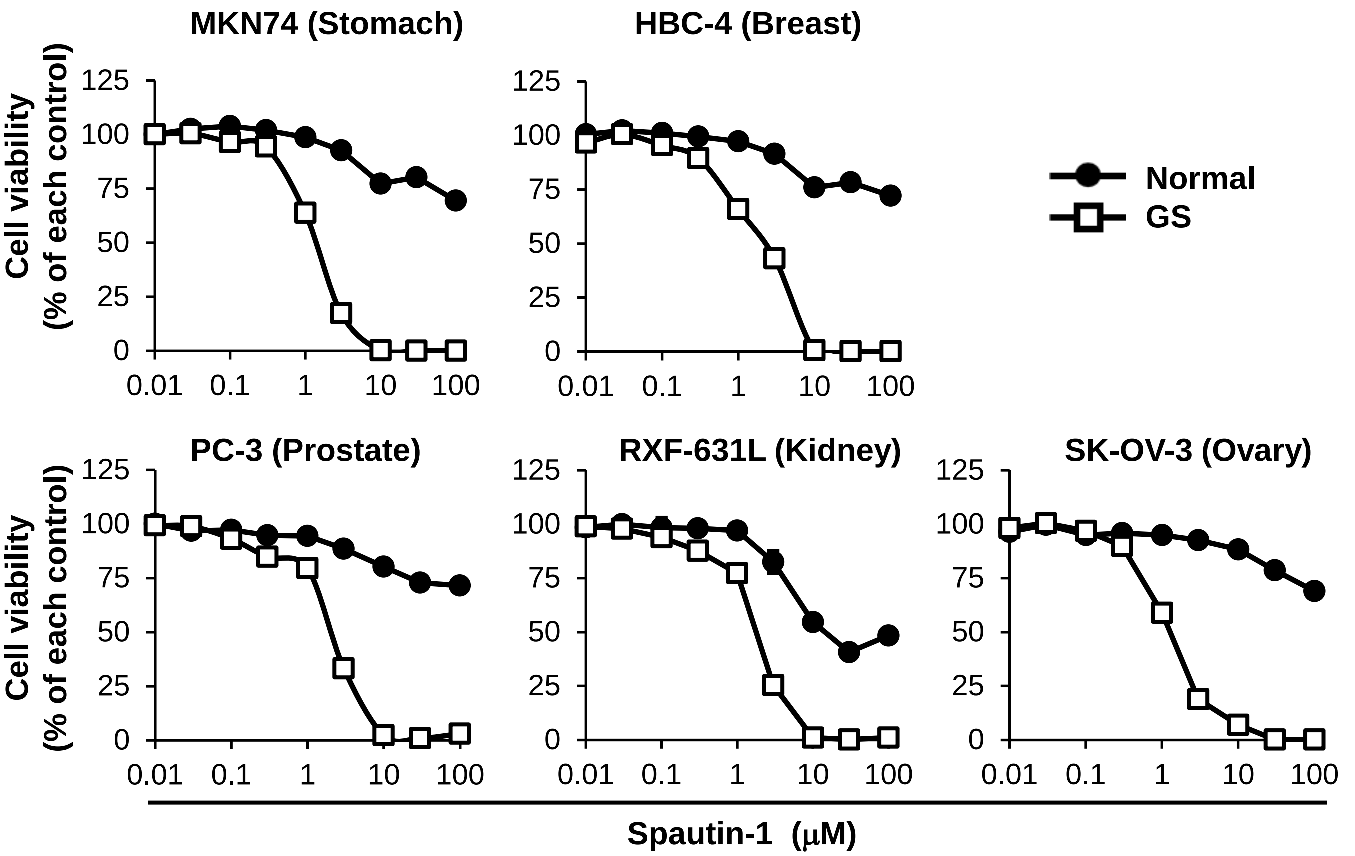

Supplementary Figure S1. Spautin-1 inhibits cancer cell survival in glucose-starved conditions.

Each cancer cell line was plated at 5 or 10 x 10^3 cells/ well in 96 well plate. After 24 h incubation, these cell lines were treated with vehicle (DMSO) or spautin-1 (0.01 – 100 μM) under normal or glucose starvation (GS) conditions for 72 h. Cell viability was determined by CellTiter-Glo Luminescent Cell Viability Assay. Data are shown as mean ± SD (*n*=3).

Supplementary Figure S2

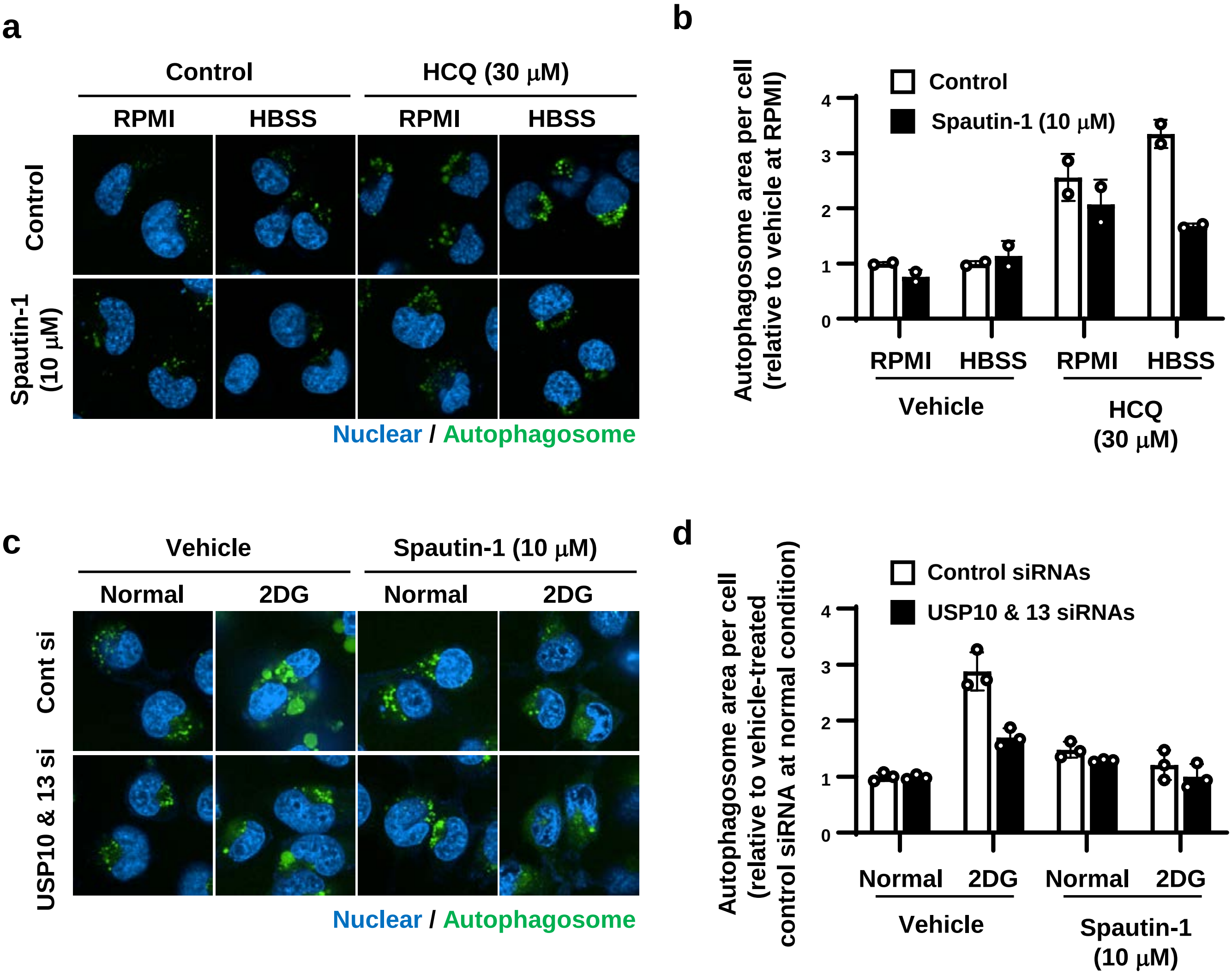

Supplementary Figure S2. Spautin-1 and silencing of USP10 and USP13 suppresses autophagosome formation.

(**a**) HT1080 cells were treated with spautin-1 (10 μM) in the presence or absence of HCQ (30 μM) under RPMI1640/ 10%FBS medium or HBSS for 4 h. The cells were stained with Hoechst33342 and CYTO-ID green detection reagent 2 for 30 min. Autophagosomes were observed using a 40x water objective lens by Operetta CLS (Perkin Elmer). (**b**) Relative autophagosome area per cell (nine fields per well) was estimated with Harmony high-content analysis software(Version number 4.9, Perkin Elmer). Data are shown as mean ± SD (*n*=2). (**c**) HT1080 cells were transfected with control siRNAs or combination of USP10 and USP13 siRNAs. After 48 h incubation, the cells were treated with vehicle or spuatin-1 (10 μM) under normal (glucose-rich) or 2DG (10 mM)-stressed conditions for 16 h. Furthermore, HCQ (30 μM) was added to the conditioned medium for 4 h to stop autophagic flux and then stained with Hoechst33342 and CYTO-ID green detection reagent 2 for 30 min. Autophagosomes were observed using a 40x water objective lens by Operetta CLS (Perkin Elmer). (**d**) Relative autophagosome area per cell (nine fields per well) was estimated with Harmony high-content analysis software(Version number 4.9, Perkin Elmer). Data are shown as mean ± SD (*n*=3).

Supplementary Figure S3

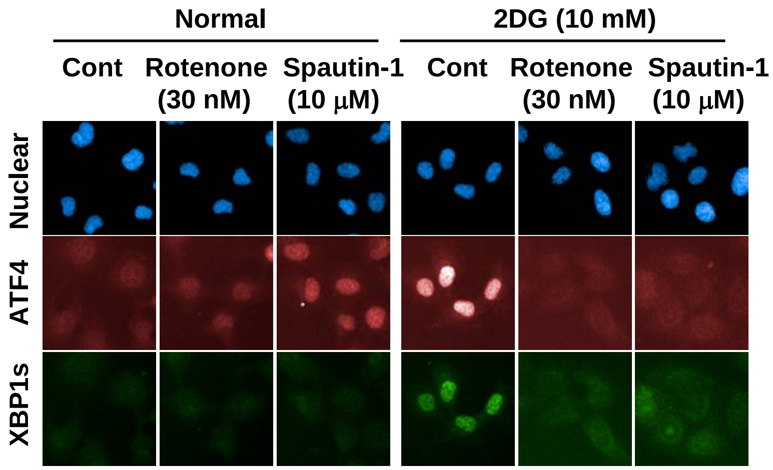

Supplementary Figure S3. Rotenone inhibits ATF4 and XBP1s induction in 2DG-stressed conditions.

Effects of rotenone (30 nM) on nuclear ATF4 and XBP1s induction under 2DG-stressed conditions were visualized using the Operetta CLS. Blue, red, and green fluorescent signals indicate nuclei, ATF4, and XBP1s, respectively.

Supplementary Figure S4

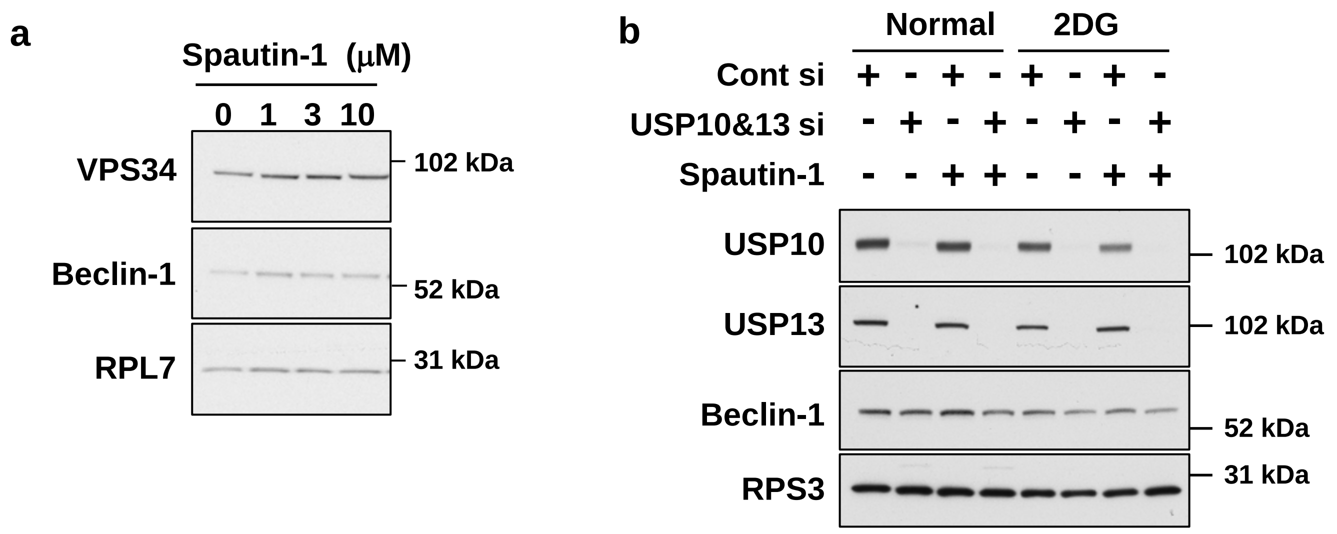

Supplementary Figure S4. Spautin-1 failed to VPS34 and Beclin-1 under normal and 2DG-stressed conditions in HT1080 cells.
(**a**) HT1080 cells were treated with spautin-1 (0, 1, 3, 10 μM) for 12 h under normal (glucose-rich) conditions. VPS34 and beclin-1 levels in HT1080 cells were determined by western blotting. RPL7 was used as an internal control. (**b**) HT1080 cells were transfected with control siRNAs or combination of USP10 and USP13 siRNAs and cultured for 48 h. The cells were treated with spautin-1 (10 μM) under normal or 2DG (10 mM)-stressed conditions for 18 h. Changes in USP10, USP13, and beclin-1 levels were determined by western blotting. RPS3 was used as an internal control.

Supplementary Figure S5

Original blot images in Figure 1b

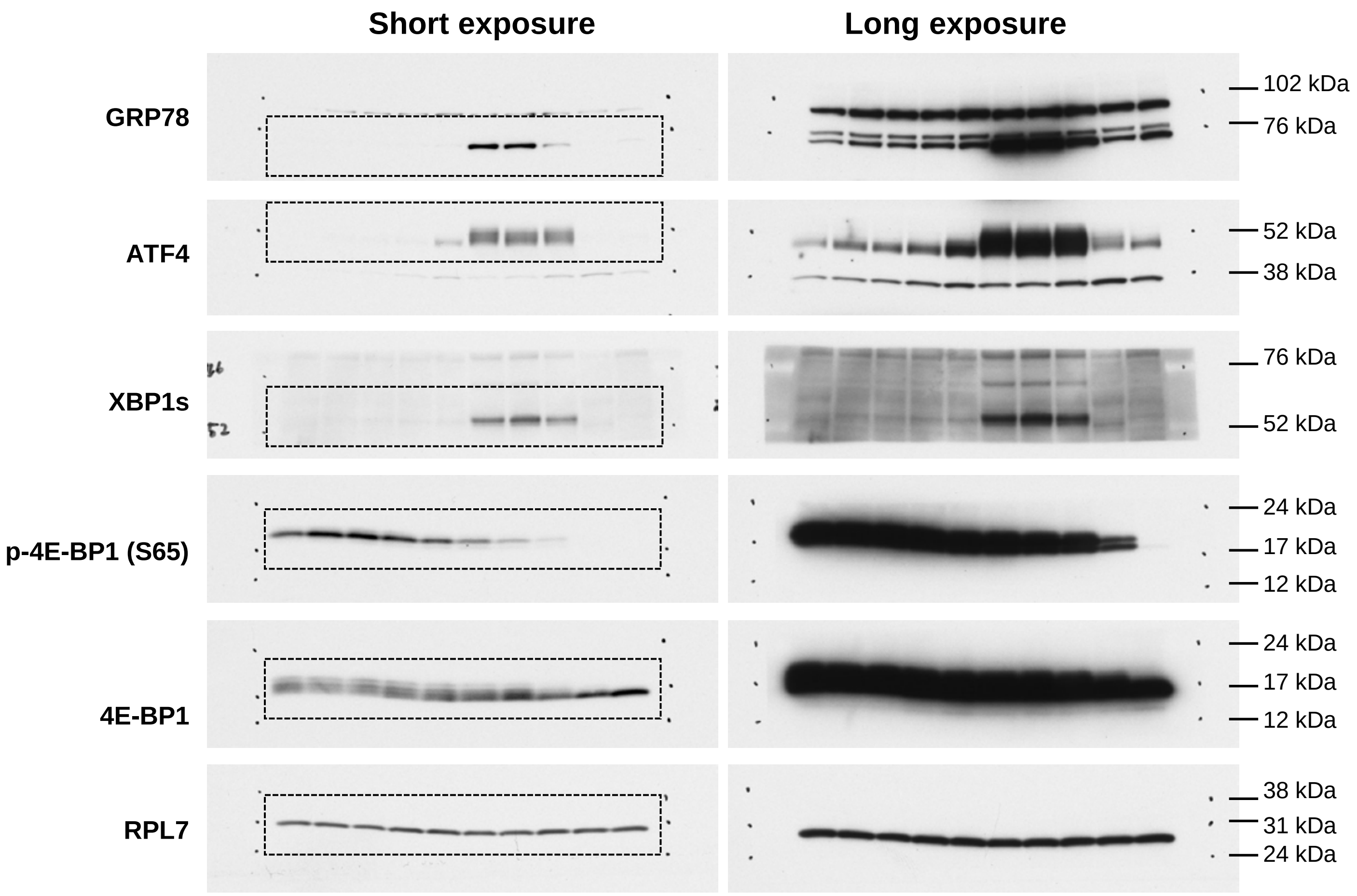

Supplementary Figure S5
Before incubation with each primary and secondary antibody, nitrocellulose membranes were cut with at least one molecular weight marker, according to Full range rainbow molecular weight markers (RPN800E, Cytiva). Original scan images for western blots in Figure 1b, 1c, 4a and supplementary Figure S4 were acquired with X-OMAT 2000 film processor (Kodak) or Amersham ImageQuant 800 (Cytiva). Two types of exposure (short and Long) per each image were provided. Dashed lines indicate areas presented as figures and supplementary figures.

Supplementary Figure S5 (Continued)

Original blot images in Figure 1c

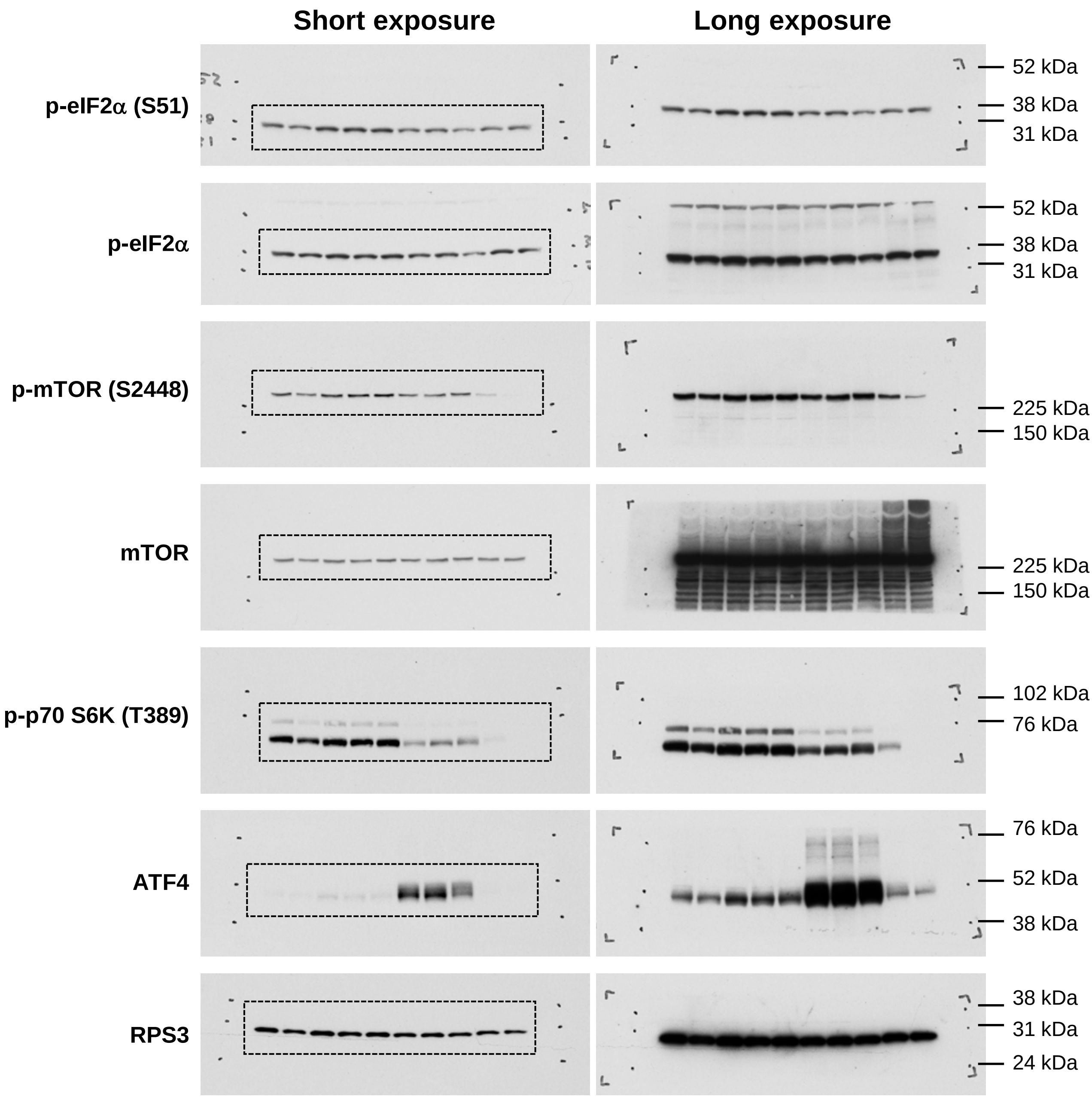

Supplementary Figure S5 (Continued)

Original blot images in Figure 4a

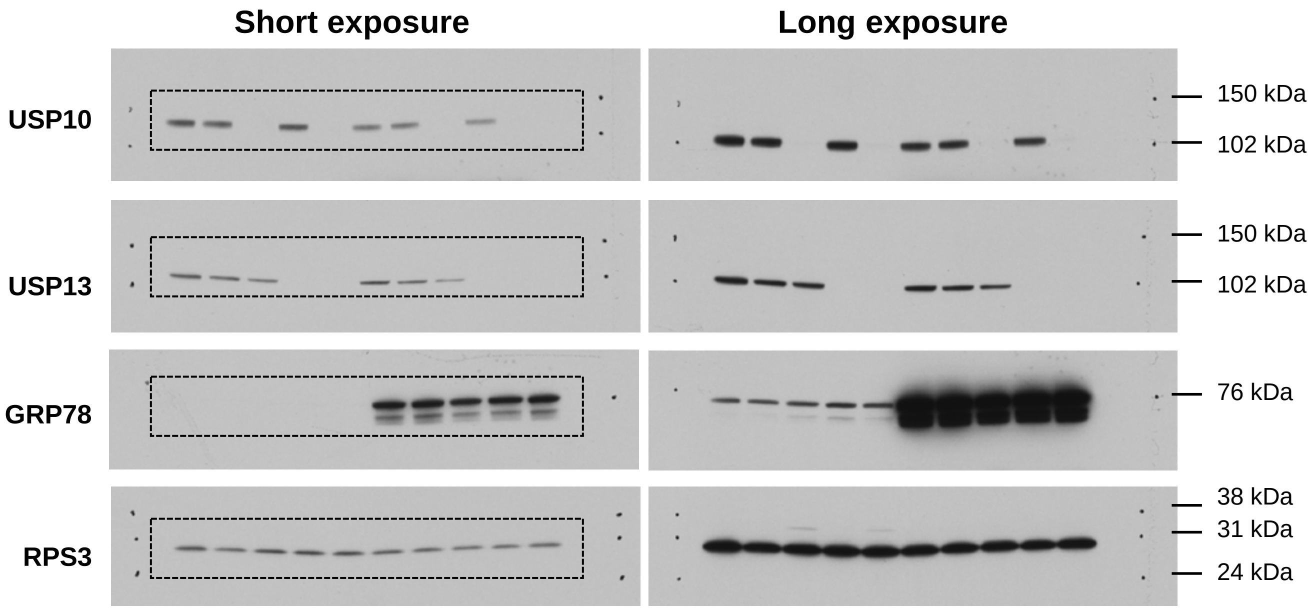

Supplementary Figure S5 (Continued)

Original blot images in Supplementary Figure S4a

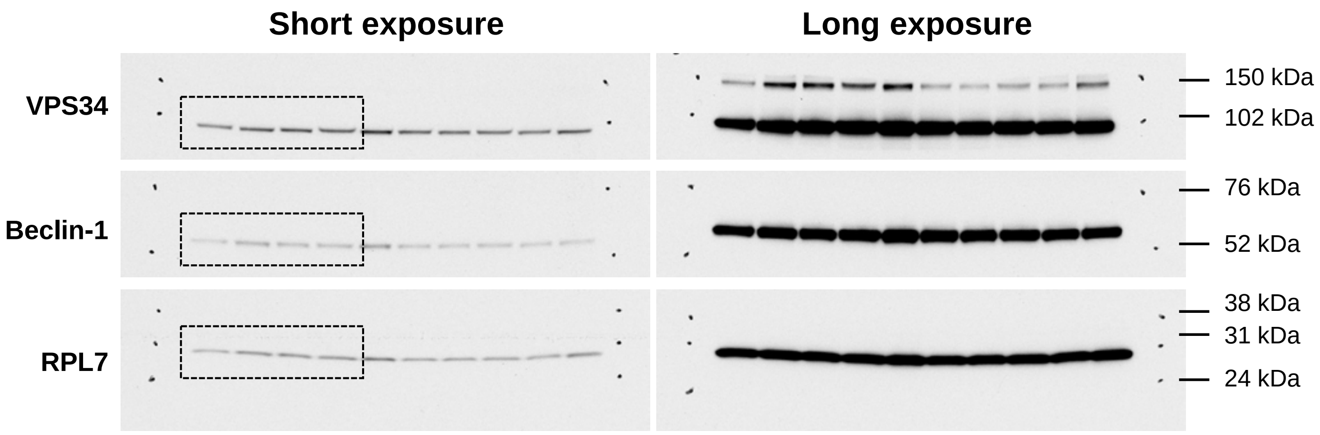

Original blot images in Supplementary Figure S4b

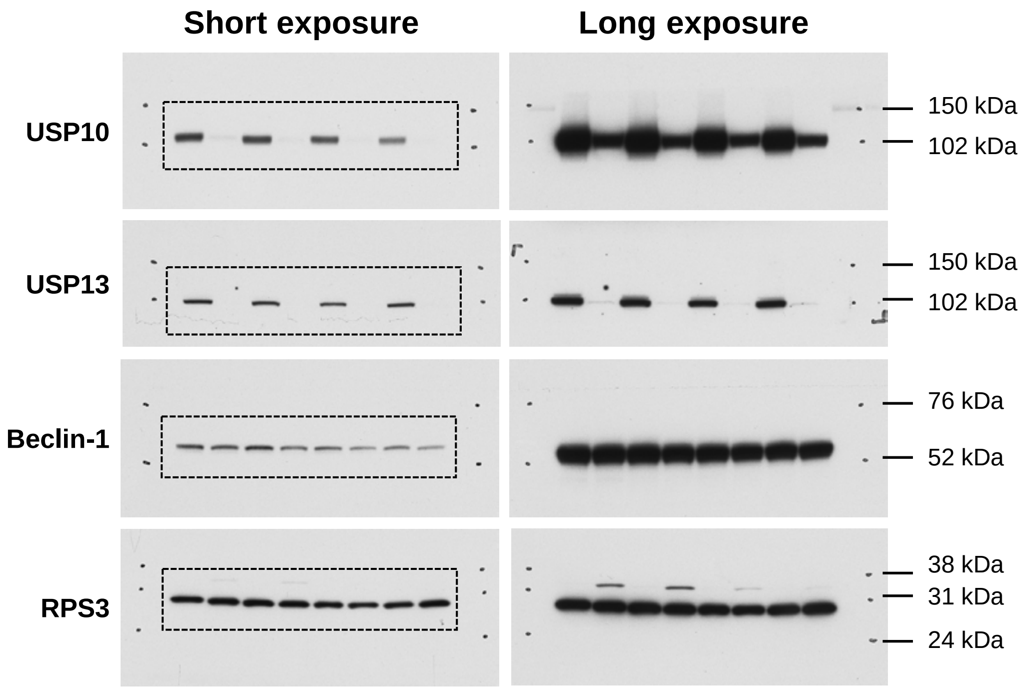

Supplement: Supplementary file 1 — Supplementary Information 1. [file 41598_2022_15673_MOESM1_ESM.pdf]
